# Remote Ischemic Preconditioning Does Not Alleviate Post–Electroconvulsive Therapy Cognitive Dysfunction

**DOI:** 10.1097/YCT.0000000000001150

**Published:** 2026-09-10

**Authors:** Ravitej Bhat, Venkatapura J. Ramesh, Jagadisha Thirthalli, Channaveerachari Naveen Kumar, Kadarapura Nanjundaiah Gopalakrishna

**Affiliations:** * Department of Anaesthesiology, Kasturba Medical College, Manipal, Manipal Academy of Higher Education, Manipal; Departments of † Neuroanaesthesia and Neurocritical Care; ‡ Psychiatry, National Institute of Mental Health and Neurosciences (NIMHANS), Bengaluru, Karnataka, India

**Keywords:** RIPC, electroconvulsive therapy, cognition, schizophrenia

## Abstract

**Objective:**

This study aimed to evaluate the efficacy of remote ischemic preconditioning in decreasing post–electroconvulsive therapy-induced cognitive dysfunction.

**Methods:**

This was a randomized controlled clinical trial in patients with schizophrenia. The patients recruited in the study were subjected to RIPC or sham RIPC. RIPC or sham RIPC was given before each ECT, and the patient was followed up with six ECTs. The cognitive and memory assessments before and after ECT within and between the groups were compared via the Hindi Mental State Examination (HMSE), Battery for Electroconvulsive Therapy-Related Cognitive Deficits (B4ECT ReCoDe), Clinical Global Impression—Severity Index (CGI-SI), and PGI Memory Scale (PGI).

**Results:**

The sociodemographic and clinical profiles were similar between the groups. The HMSE (*P* = 0.824), B4ECT ReCoDe (*P* = 0.225), PGI (*P* = 0.111), and CGI-S (*P* = 0.776) scores were not significantly different between the groups.

**Conclusions:**

Remote ischemic preconditioning was not successful in decreasing post–cognitive dysfunction in patients with schizophrenia who were receiving electroconvulsive therapy. However, it was safe and feasible in ECT patients and did not have any significant effect on the primary disease.

Electroconvulsive therapy (ECT) is one of the most effective modalities for the treatment of psychiatric disorders.^1^ However, post-ECT cognitive dysfunction is an important cause of functional impairment. The incidence of post-ECT cognitive adverse effects has varied from 9% to 55% in various studies.^2–5^ A multitude of mechanisms have been implicated in the cause of post-ECT cognitive dysfunction, including glutamate excitotoxicity, inflammation, and oxidative stress.^6,7^ Preventive interventions on the basis of these mechanisms have subsequently been attempted but with limited success.

Ischemic preconditioning has been used in cardiac bypass surgeries to prevent increases in infarct size.^8^ In general, ischemic preconditioning disrupts the vascular supply for a brief period to cause sublethal ischemia.^9^ However, this invasive approach proves to be clinically impractical when other organ systems and procedures are involved. This led to the development of a novel noninvasive way of protecting organs from ischemic injury known as remote ischemic preconditioning (RIPC).^10^

RIPC works on the principle of causing brief episodes of nonlethal ischemia in limbs far from the organ, which results in the release of protective factors that act on distant organs to prevent cell death, thereby diminishing the severity of ischemic injury.^11^ RIPC's mechanisms of action, including antioxidant effects, gene modification, and anti-inflammatory properties,^12^ align well with the pathophysiological processes implicated in ECT-induced cognitive dysfunction.

Studies of RIPC in cardiac surgery have demonstrated its potential for neurocognitive protection.^13–17^ Animal models and human trials have shown that RIPC can reduce cognitive decline following cardiac procedures, suggesting its potential utility in other contexts where cognitive function may be at risk. However, despite these promising findings in cardiac surgery, no studies have investigated the effects of RIPC on cognitive outcomes following ECT.

Given the pressing need for effective interventions to prevent ECT-related cognitive impairments, this study aims to evaluate the role and efficacy of remote ischemic preconditioning in decreasing post–electroconvulsive therapy-induced cognitive dysfunction. We hypothesized that administering RIPC before ECT would preserve cognitive function compared with sham preconditioning.

## METHODOLOGY

### Study Design and Participants

This was a prospective, single-blind, randomized controlled trial conducted over a period of 2 years in NIMHANS. The study was approved by the institutional ethics committee and was registered in the Clinical Trial Registry of India (CTRI/2017/04/008358). Study participants were selected randomly on the basis of the inclusion and exclusion criteria. Patients aged 18 to 65 years of either sex, diagnosed with schizophrenia, schizoaffective disorder, or unspecified nonorganic psychosis according to the International Classification of Disease-10 (ICD-10) criteria and scheduled to undergo electroconvulsive therapy (ECT), were eligible for inclusion. The diagnosis of these disorders is made clinically by independent psychiatric evaluation by 2 qualified psychiatrists in all cases. The exclusion criteria included upper limb cellulitis; ulcers; peripheral vascular disease; comorbid cardiac, renal, hepatic, pulmonary, or neurological conditions; mental retardation; and substance use disorders (except nicotine).

### Randomization and Blinding

>Eligible participants were randomly assigned at a 1:1 ratio to either the remote ischemic preconditioning (RIPC) group (Group A) or the sham RIPC group (Group B) via a computer-generated random number sequence. Allocation concealment was achieved via sealed opaque envelopes. The participants were blinded to their group assignment.

### Ethical Consideration

The research was conducted in strict accordance with the Declaration of Helsinki and Good Clinical Practice standards, ensuring that participants' rights and well-being were prioritized. Approval was obtained from the institutional ethics committee, which oversaw and assessed the study. Informed consent was obtained from the patients if they consented to their ECT. The informed consent procedure was conducted by either the main investigator or coinvestigators, who thoroughly explained all facets of the study, including potential advantages and risks. Patients received a detailed information sheet and had the chance to inquire about any concerns they had directly with the investigator. If the patients were not in a state of giving consent for ECT, then the consent for the research was obtained from the next of kin who had provided consent for ECT; in the latter case, informed consent was obtained from the patients as soon as they were good enough to provide consent.

### Interventions

#### Group A (RIPC Group)

Participants received three 5-minute cycles of upper limb ischemia with 5-minute intervals of reperfusion in between, which were administered before each ECT session. The cuff was inflated to a pressure 30 mm Hg above the systolic blood pressure.^18,19^

#### Group B (Sham RIPC Group)

Participants received a sham RIPC procedure with the cuff inflated to 30 mm Hg for 5 minutes, followed by 5 minutes of deflation. Three such cycles were administered over a period of 30 minutes.^18,19^ The sham RIPC procedure was designed to create a sensory experience similar to that of the RIPC group, utilizing pressure that mimics the sensation of inflation while avoiding actual ischemic effects. This design is pivotal for ensuring that participants remain unaware of their group allocation.

The procedure was terminated if the patient reported any severe discomfort in the upper limb. At the end of the RIPC protocol, the limb was evaluated clinically for signs of any ischemic damage and documented in a checklist.

### ECT Administration

This study employed electroconvulsive therapy (ECT) via the Niviqure VR device manufactured by Niviqure Meditech Pvt Limited in Bengaluru (Bangalore). The electrical stimulus parameters included a constant current of 800 mA, with a frequency ranging between 75 and 125 pulses per second. The pulse width was 1 millisecond.

On the day of the procedure, a pulse oximeter, an ECG, and NIBP were connected to the patient. The baseline vital signs, such as pulse rate and blood pressure, were recorded. The patients' vital signs were recorded at 0 minutes, 10 minutes, 20 minutes, and 30 minutes during RIPC. The patient then underwent ECT under general anesthesia according to a standardized anesthesia protocol. The patient was initially oxygenated for 3 minutes, and 5 mg/kg i.v. thiopentone was given. After loss of consciousness, 0.01 mg/kg i.v. atropine and 1 mg/kg i.v. succinylcholine were administered. After adequate muscle relaxation was confirmed, the patient received bitemporal or bifrontal bilateral brief pulse square wave ECT as prescribed by his/her psychiatric team. The RIPC or sham RIPC protocol was applied before each consecutive ECT session up to the sixth ECT or the last ECT session, whichever occurred earlier.

#### Study Procedure

Initially, eligible participants were screened on the basis of inclusion and exclusion criteria, after which informed consent was obtained. During the baseline visit, patients' demographic information and baseline vital signs, such as pulse rate and blood pressure, were recorded. Cognitive function was assessed via the Hindi Mental State Examination (HMSE), the PGI Memory Scale, and components of the B4ECT-ReCoDe. The Clinical Global Impression-Severity (CGI-S) scale was utilized to assess the severity of symptoms. Patients were then randomly assigned to receive either RIPC or sham RIPC. On the day of the procedure, a pulse oximeter, ECG, and noninvasive blood pressure (NIBP) monitor were connected to the patient. Vital signs were recorded at 0, 10, 20, and 30 minutes during the RIPC or sham RIPC application. The patient then underwent ECT under general anesthesia according to a standardized anesthesia protocol, which included preoxygenation for 3 minutes, followed by intravenous thiopentone at a dose of 5 mg/kg. After loss of consciousness, atropine (0.01 mg/kg i.v.) and succinylcholine (1 mg/kg i.v.) were administered. Once adequate muscle relaxation was confirmed, the patient received either bitemporal or bifrontal bilateral brief pulse square wave ECT, as prescribed by the treating psychiatry team. The RIPC or sham RIPC protocol was applied before each consecutive ECT session, up to the sixth ECT session or until the last ECT session, whichever occurred first. Cognitive assessments were performed immediately after the sixth ECT or the last ECT, whichever occurred first in both groups. Throughout the study, any adverse effects were monitored closely.

### Outcome Measures

The primary outcome was cognitive function, which was assessed via the following tools:

### Hindi Mental Status Examination (HMSE)

The Hindi Mental State Examination (HMSE)^20^ is an Indian adaptation of the Mini Mental Status Examination (MMSE) for epidemiological investigations of dementia. The scale has been modified to suit the Indian population.

### PGI Memory Scale

The PGI memory test is the modified Indian version of the Weshsler memory scale.^21^ It consists of a comprehensive battery of 10 subtests. The language is simple and does not depend unduly on intelligence.

### Battery for ECT-Related Cognitive Deficits (B4ECT-ReCoDe)

This test was developed in India to assess the cognitive deficits associated with ECT.^22^ It contains 10 components. We used the autobiographical memory questionnaire, the digit symbol substitution test, and the letter number sequencing part of the battery.

### Clinical Global Impression—Severity Index Scale (CGI-SI)

This scale is used to assess the severity of symptoms in psychiatric patients.^23^ The CGI-SI is rated on a 7-point scale (1, normal; 7, most severely ill). When measured periodically, it can be used to evaluate the response to treatment.

Cognitive assessments were performed before the first ECT and after the sixth ECT or the last ECT, whichever was earlier, within 24 to 48 hours after administering the ECT.

### Sample Size

The required sample size for this randomized controlled trial was calculated to detect a moderate effect size (Cohen's *d* = 0.637). Previous studies using similar interventions, although for different indications,^17,18^ reported moderate effect sizes, ranging from 0.55 to 0.75. On the basis of this and statistical convention, where an effect size of 0.5 or more is considered moderate, we chose to detect a moderate effect size in our study. With a two-sided significance level of 0.05 and a desired power of 80%, the sample size was 80, which was 40 in each group, accounting for 10% of the dropout rates.

### Statistical Analysis

All the data are expressed as either the mean ± standard deviation or the median and interquartile range, depending on their distribution. The normality of the data was assessed via the Shapiro-Wilk test. For data that were not normally distributed, the Mann-Whitney *U* test was used to compare unpaired data, whereas the Wilcoxon signed-rank test was applied for paired data. For normally distributed data, unpaired and paired *t* tests were performed. The level of statistical significance was set at *P* < 0.05. All the data were analyzed via SPSS version 16.

## RESULTS

**FIGURE 1:**
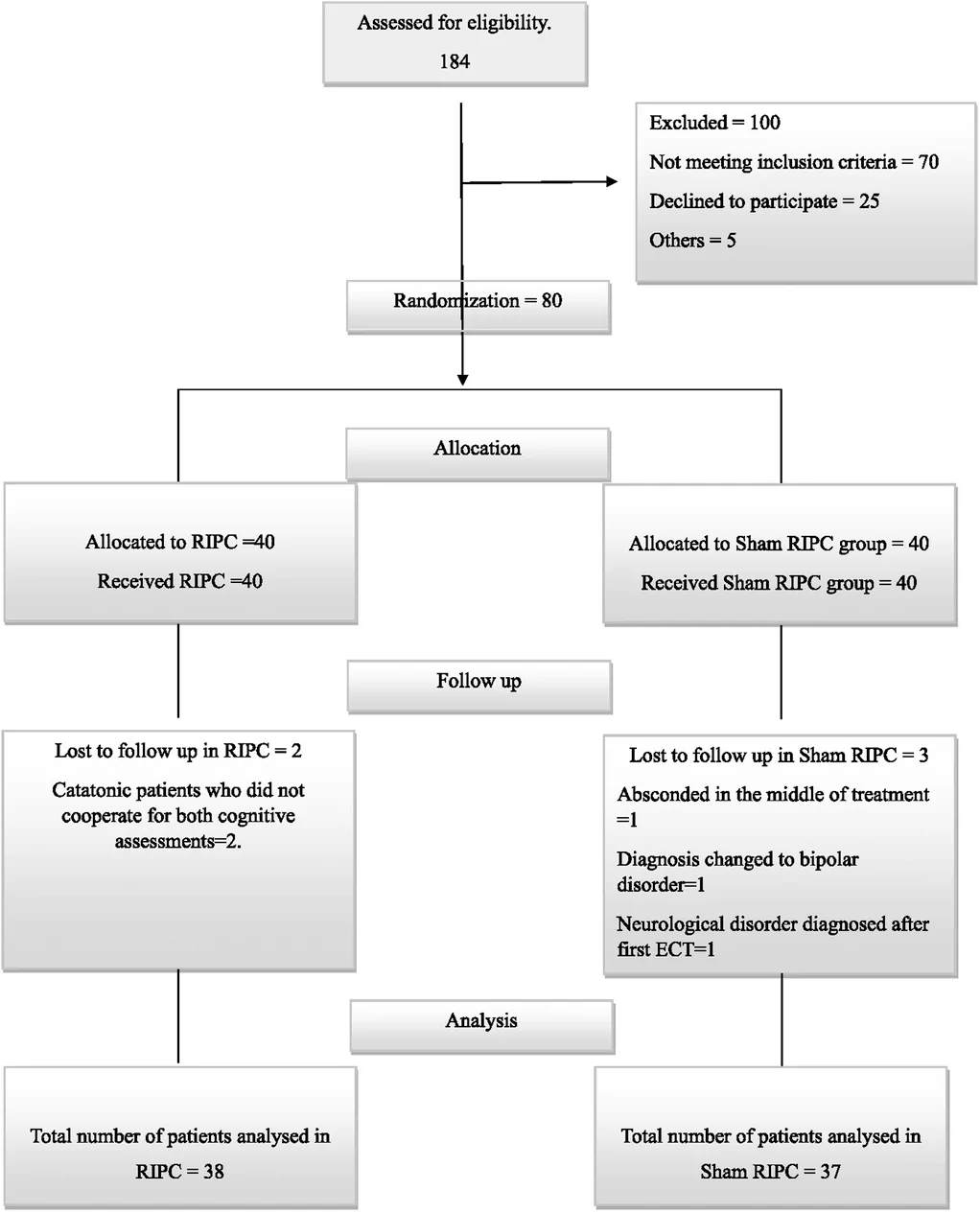
CONSORT flow diagram showing patient recruitment and allocation to the remote ischemic preconditioning (RIPC) and sham RIPC groups.

Details regarding patient recruitment are given in the CONSORT flowchart in Figure 1. Among the 184 patients initially screened for the study, 104 were not recruited for the study. Of these, 74 did not meet the specific criteria for the study, 25 chose not to participate, and the remaining 5 were excluded for other reasons. The remaining 80 participants were randomized, with 40 allocated to the RIPC group and the other 40 allocated to the sham RIPC group. In the RIPC group, two catatonic patients did not cooperate for either cognitive assessment and were excluded from the analysis. Similarly, three patients in the sham RIPC group did not complete the study (one dropped out in the middle of treatment without providing any reason, one had a diagnosis changed to bipolar disorder, and one was diagnosed with a neurological disorder after the first ECT). Hence, 38 patients in the RIPC group and 37 patients in the sham RIPC group completed the study. All the patients included in the study were inpatients.

The sociodemographic and clinical profile of the sample is shown in Table 1. There was no statistically significant difference between the two groups with respect to age, sex, literacy, marital status, socioeconomic status, or routine investigation parameters (Table 1).

**TABLE 1:** Sociodemographics and Clinical Details Between the Two Groups

Parameters	RIPC Group	Sham RIPC Group	*P* Value
Age (y) (mean ± SD)	33.4 ± 9.45	32.8 ± 8.26	0.610
Weight (kg) (mean ± SD)	57.18 ± 14.87	58.45 ± 11.67	0.464
Sex (F/M) (%)	55/45	35/65	0.072
Marital status (divorced/married/single) (%)	10/32.5/57.5	7.5/40/52.5	0.762
Duration since last ECT (mean ± SD)	26.79 ± 17.14	23.5 ± 13.27	0.758
Education (graduate/illiterate/postgraduate/school) (%)	22.5/17.5/7.5/52.50	20/5/7.5/67.5/0	0.310
Employment (employed/unemployed) (%)	15.85	12.5/87.5	0.745
ASA grading (1/2) (%)	85/15	92.5/7.5	0.288
Socioeconomic status (middle class/poor/upper middle class) (%)	25/72.5/2.5	32.5/67.5/0	0.481
Diagnosis (catatonic schizophrenia/paranoid schizophrenia/schizoaffective disorder/undifferentiated schizophrenia) (%)	7.5/67.5/0/25	7.5/57.5/5/30	0.475
Past history of ECT (no/yes) (%)	65/35	77.5/22.5	0.215
Number of ECT received during study period (mean ± SD)	5.25 ± 0.97	5.12 ± 0.82	0.576

The most common indications for ECT were first-line therapy and augmentation of the drug response, which were equally represented in the 2 groups. The time since last ECT in those with a prior history was 6 months or more, and the duration and number of patients with a past history of ECT were not significantly different between the 2 groups (Table 1). ECT-related cognitive deficits are not known to last for more than a few weeks.^24^ Hence, these parameters will not have any effect on the final outcome of the study.

**FIGURE 2:**
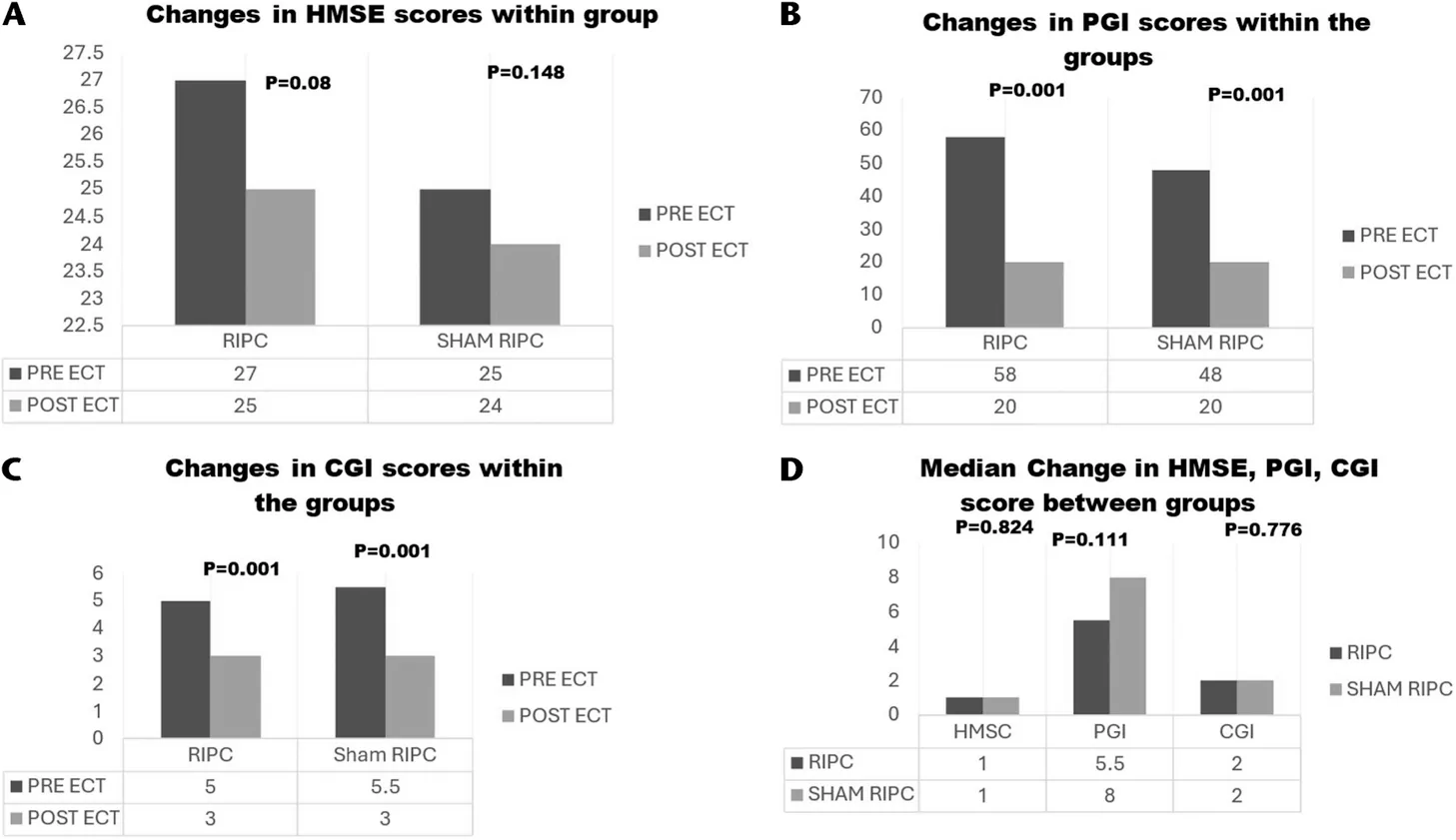
Cognitive assessment scores within and between the RIPC and sham RIPC groups. This figure consists of four panels (A–D) showing the results of different cognitive assessments. A, Hindi Mental State Examination (HMSE) scores: this panel shows the HMSE scores before and after ECT for both the RIPC and sham RIPC groups. The lack of a significant change in scores suggests that general cognitive screening results remained stable after ECT in both groups. B, PGI Memory Scale scores: this panel displays the PGI Memory Scale scores before and after ECT for both groups. A decrease in scores indicates a decline in memory function. The graph shows a significant within-group decrease in both the RIPC and sham RIPC groups. C, Clinical Global Impression-Severity Index (CGI-SI) scores: this panel presents both groups' CGI-SI scores before and after ECT. A decrease in score indicates improvement in clinical severity. Both groups showed significant improvement. D, Comparison of changes in HMSE, PGI, and CGI scores: this panel compares the changes in all three measures between the RIPC and sham RIPC groups, visually representing the lack of significant differences between groups across these measures.

The cognitive assessment scores by HMSE were not statistically significant when the scores were assessed both within and between the groups (Fig. 2A and D). A decreasing score indicates decreased cognitive dysfunction.

The scores of the PGI memory scale were not significantly different between the 2 groups (Fig. 2D). There was a significant difference within the group (Fig. 2B) in both groups (*P* < 0.05, significance).

**FIGURE 3:**
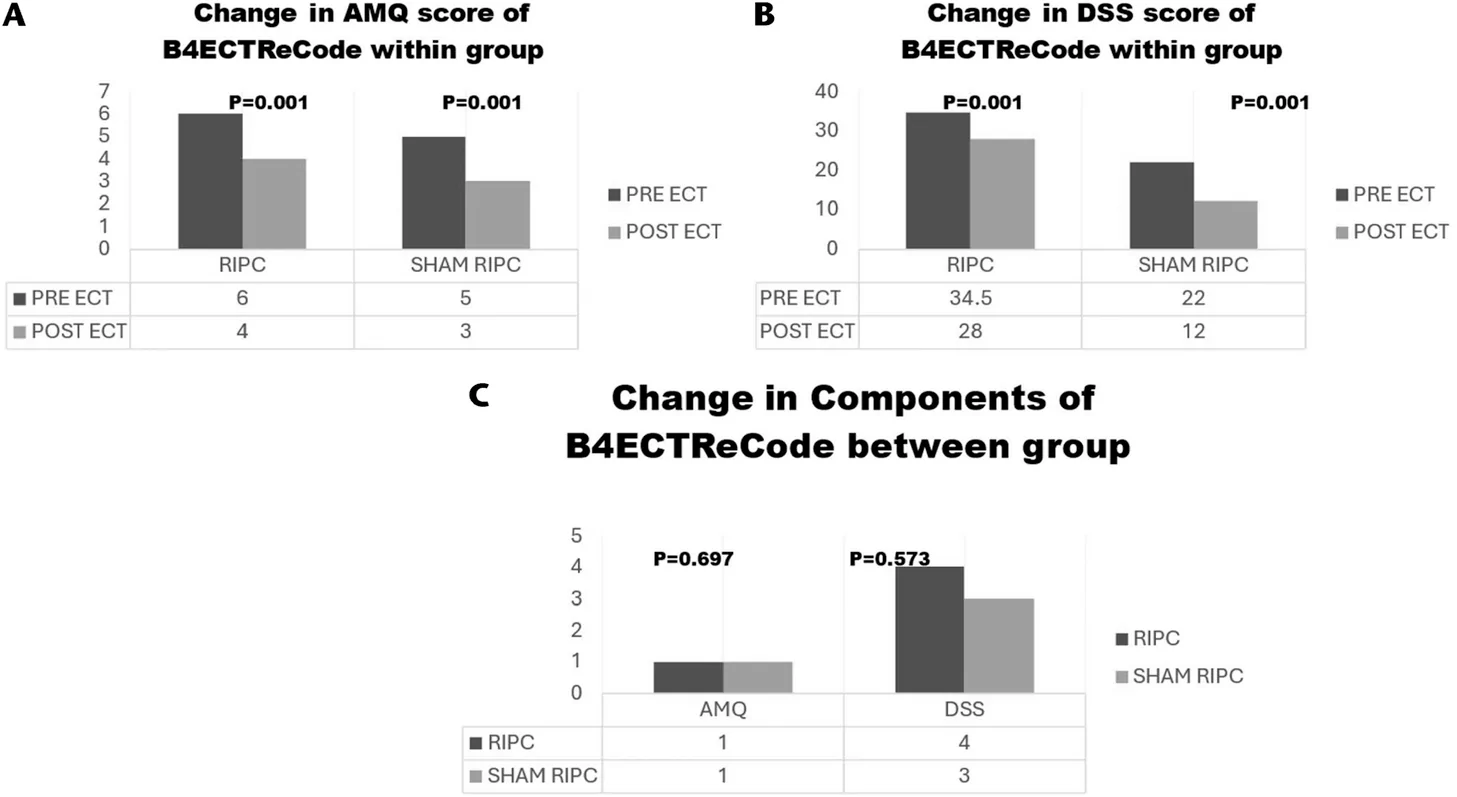
Changes in B4ECTReCode component scores. This figure consists of three panels (A–C) showing the results of the Battery for ECT-Related Cognitive Deficits (B4ECTReCode). A, Changes in AMQ scores within groups. B, Changes in DSS score within groups. C, Changes in scores for three B4ECTReCode components (AMQ, DSS, and LNS) before and after ECT in the RIPC group and the sham RIPC group. For all panels, the AMQ (Autobiographical Memory Questionnaire) and DSS (Digit Symbol Substitution test) show significant decreases in scores after ECT, indicating cognitive decline. LNS (Letter Number Sequencing) scores were low at baseline and remained unchanged after ECT in both groups. The figure shows that, although there were significant within-group changes, there were no significant differences between the RIPC and sham RIPC groups.

The three important cognitive components of the B4ECTReCode did not differ between the RIPC and sham RIPC groups, as shown in Figure 3C. AMQ (Fig. 3A) and DSS (Fig. 3B) significantly decreased in score from pre-ECT to post-ECT, whereas LNS was very low, and a median of zero was obtained for both groups, and the score remained the same after ECT.

The clinical severity scale, as assessed by the CGI-S, significantly improved within each group (Fig. 2C), but it was comparable between the 2 groups (*P* = 0.776) and was not significantly different between the groups, as shown in Figure 2D.

The intra-ECT parameters, such as the anesthetic regimen, seizure duration, type of electrode placement, and charge, were comparable between the two groups (Table 2).

**TABLE 2:** Comparison of ECT Related Anesthetic Dose, Seizure Duration, and Stimulus Intensity (Charge) Between the Two Groups

Parameters	RIPC Group (Mean ± SD)	Sham RIPC Group (Mean ± SD)	*P* Value
Thiopentone (mg)	137.5 ± 25.94	138.13 ± 25.31	0.972
Succinycholine (mg)	23.13 ± 6.67	23.63 ± 6.89	0.890
Atropine (mg)	0.6 ± 0.0007	0.6 ± 0.0002	1.000
Charge (mC)	160.5 ± 77.33	156 ± 63.44	0.896
Seizure duration (s)	49.55 ± 11.44	53.43 ± 10.76	0.092

Hemodynamic variables such as heart rate and mean arterial pressure were measured at 4 time points during a single RIPC/sham RIPC session and over the subsequent 6 such sessions. There was no significant variation over both time points (data available on request).

## DISCUSSION

Electroconvulsive therapy (ECT) remains one of the most important therapeutic modalities in the treatment of schizophrenia. This study examined the efficacy of preventing post-ECT cognitive dysfunction by applying noninvasive therapy in the form of remote ischemic preconditioning (RIPC). The findings of this study did not demonstrate a significant difference in cognitive outcomes between the RIPC group and the sham RIPC group, as assessed by various cognitive assessment tools, such as the HMSE, PGI Memory Scale, and B4ECT-ReCoDe.

The anti-excitotoxic, antioxidative, and anti-inflammatory properties of RIPC^25,26^ mirror the neurobiological mechanisms underlying ECT-induced cognitive decline, such as glutamate excitotoxicity, oxidative stress, NMDA receptor activation, and inflammation.^27,28^ However, the lack of a significant effect of RIPC on post-ECT cognitive function in this study contradicts the hypothesized benefits of the proposed mechanisms of RIPC.

The vulnerability of the brain to damage during procedures such as preconditioning makes RIPC an attractive potential protector. Studies have shown that RIPC successfully improves outcomes after various brain injuries, such as secondary brain injury, spinal cord injury, neurodegenerative diseases, stroke, and small vessel disease.^29–31^ Only a few studies have investigated the effects of RIPC on cognitive parameters. However, the impact of RIPC on cognitive function remains unclear. Whereas some studies reported reduced cognitive decline after RIPC in procedures such as cardiopulmonary bypass, colon surgery, and knee arthroplasty,^13–18,32–35^ others reported mixed results. A pilot study on stroke patients showed promising results for RIPC, indicating that it is safe and feasible. However, the study concluded that larger trials are needed to definitively determine its effectiveness in improving cognitive function.^36^ Our study is the first to investigate the efficacy of RIPC in preventing post-ECT cognitive decline. Unfortunately, post-ECT RIPC did not yield positive results. This aligns with a recent review on RIPC and cognitive function after heart surgery. This meta-analysis of five clinical trials revealed that RIPC did not improve cognition following cardiopulmonary bypass (CPB)–assisted heart surgery.^14^

ECT-induced cognitive dysfunction is one of the important causes of hesitation in prescribing and undergoing ECT as a therapy for psychiatrists and patients, respectively. Objective measures revealed memory loss to be relatively short term (6 weeks) posttreatment, whereas subjective measures reported amnesia to be more persistent (6 months post-ECT).^37^ Patients admitted from the six psychiatry units in our institution were recruited for our study. Augmentation of drug therapy due to the need for an urgent response and first-line treatment, e.g., catatonia, suicide, and aggression, were the most common indications for ECT, which was similar to the findings of previous studies.^38–40^ All patients in our study underwent the standard ECT protocol with no complications (e.g., hypoxic episodes, cardiovascular complications, etc.) during or after ECT that could have affected outcomes. The clinical severity scale, as assessed by the CGI-SI, showed a significant improvement within each group, indicating that ECT was effective in reducing the severity of psychiatric symptoms in both the RIPC and sham RIPC groups. However, the extent of improvement in clinical severity was comparable between the two groups, with no statistically significant difference between them. The fact that there was no difference between the RIPC and sham RIPC groups implies that RIPC did not have any influence, positive or negative, on the worsening of symptom severity following ECT compared with the sham group. Hence, RIPC was safe and feasible for patients with schizophrenia after ECT.

Memory was the primary focus of tools such as the Hindi Mental State Examination (HMSE), PGI Memory Scale, and components of the B4ECT ReCoDe battery that were utilized in the assessment of cognitive functions in our study. These scales are specifically designed for the Indian population. Within-group analyses indicated that, although general cognitive screening using HMSE remained unchanged, ECT had a detrimental impact on memory functioning in both groups, as evidenced by the PGI Memory Scale and B4ECT ReCoDe scores. The autobiographical memory questionnaire (AMQ) and digital symbol substitution test (DSS) components of the B4ECT ReCoDe battery exhibited significant cognitive decline after ECT therapy, whereas the Letter Number Sequencing (LNS) scores were low even at baseline and did not differ significantly after the sixth ECT session in either group. Hence, our findings corroborate previous studies showing that ECT can cause significant cognitive impairment, particularly in the domains of memory.

One potential explanation for the lack of significant findings could be related to the intensity of the ischemic preconditioning stimulus. Although the duration and frequency of RIPC were standardized in this study, the adequacy of the ischemic stimulus intensity was not assessed. Some studies have suggested that there may be a narrow range of ischemic tolerance where cellular apoptosis is inhibited, and an inadequate intensity of the preconditioning stimulus could potentially render RIPC ineffective.^19,41,42^ The negative results in this study could be attributed to an insufficient intensity of the ischemic stimulus for mitigating post-ECT cognitive dysfunction.

Our patients were on various drugs used for the treatment of schizophrenia. Additionally, these drugs, which act on the CNS, could have potentiated or diminished the signaling cascades involved in preconditioning and therefore affected the final outcome. However, these could not be stopped just for the sake of the study, as one of the most common indications for ECT in these patients was the augmentation of the drug response. However, all these confounding factors are equally distributed in both groups of patients, making the results of this study still valid.

The study had several strengths, including its prospective, randomized, and blinded design, as well as the use of standardized cognitive assessment tools validated for the Indian population. Additionally, the study ensured that patients did not experience any complications during or after ECT that could have affected the results, such as hypoxic episodes or cardiovascular complications.

Limitations of the study include the lack of assessment of biomarkers suggestive of neuronal injury, if any, which could have provided insights into the occurrence of neuronal damage post-ECT and the potential protective effects of RIPC. The sample size, although adequate for the primary analysis, may have limited the ability to detect smaller effect sizes. An important limitation of this study was that we did not prespecify primary and secondary outcomes in our protocol. We did not specify any outcome as a primary outcome measure. This was because we wished to assess multiple domains of cognition that are commonly affected by ECT. However, not having a primary outcome measure is problematic in the context of type-1 error (i.e., erroneously rejecting a true null hypothesis). As we did not identify any significant difference between the two groups, the impact of this drawback on the interpretation of the results is negligible. Additionally, the study focused on a specific population of patients with schizophrenia, which may limit the generalizability of the findings to other psychiatric disorders or patient populations. Not using structured interviews to establish the diagnoses is an additional limitation.

Future research could explore the potential of RIPC in combination with other pharmacological or nonpharmacological interventions for mitigating ECT-related cognitive dysfunction. Furthermore, investigating the optimal timing and duration of RIPC administration, as well as the potential dose-response relationship, could provide valuable insights. Neuroimaging studies or biomarker analyses may also help elucidate the underlying mechanisms and identify potential responders to RIPC in the context of ECT.

## CONCLUSIONS

In conclusion, although the current study did not find a significant benefit of RIPC in reducing cognitive dysfunction following ECT in patients with schizophrenia, RIPC was safe and feasible in post-ECT patients and did not have any significant effect on primary disease. This contributes to the growing body of research exploring potential strategies to minimize the adverse cognitive effects associated with this important therapeutic modality. Further research is warranted to fully understand the role and potential applications of RIPC in mitigating ECT-related cognitive impairment.
